# Duplex PCR-Based Molecular Diagnosis of Bovine Tuberculosis and Paratuberculosis: Development and Field Assessment

**DOI:** 10.3390/pathogens15101055

**Published:** 2026-10-06

**Authors:** Saurabh Gupta, Samiksha Agrawal, Swadha Pandey, Shoor Vir Singh

**Affiliations:** 1 Department of Biotechnology, Institute of Applied Sciences & Humanities, GLA University, Mathura 281406, Uttar Pradesh, India; samiksha.agrawal_phd.bio22@gla.ac.in (S.A.); shoorvir.singh@gla.ac.in or shoorvir.singhdbt@gmail.com (S.V.S.); 2 Division of Hematology & Oncology, Department of Medicine, Medical College of Wisconsin, Milwaukee, WI 53226, USA; spandey@mcw.edu

**Keywords:** zoonotic diseases, duplex PCR, *Mycobacterium bovis*, Johne’s disease, *M. paratuberculosis*, diagnostics

## Abstract

Zoonotic pathogens (*Mycobacterium bovis* and *Mycobacterium avium* subsp. *paratuberculosis*, or MAP) cause bovine tuberculosis and Johne’s disease, respectively, both of which severely affect livestock health and productivity and pose risks to human health. Accurate diagnosis is challenging due to overlapping clinical signs and genetic similarities. In the present study, we developed and preliminarily evaluated a duplex PCR assay for simultaneous detection of MTBC-associated *M. bovis* and MAP. Based on in silico primer screening and gradient PCR optimization, *IS1081* and *IS900* were selected as target sequences for the detection of MTBC-associated *M. bovis* and MAP, respectively, with an optimal annealing temperature of 60 °C. The assay was subsequently assessed using 114 blood and fecal specimens from 57 clinically suspected weak cattle under pilot field conditions, providing a preliminary proof-of-concept evaluation. Sensitivity analysis using spiked fecal samples (10^1^–10^6^ CFU/g) demonstrated a detection limit of 10^2^ colony-forming units per gram (CFU/g) for both pathogens with no cross-reactivity; amplification at 10 CFU/g was inconsistent, indicating the lower sensitivity threshold of conventional PCR. When compared with single-target *IS900* and *IS1081* PCRs, the duplex assay achieved 63.6–100% sensitivity and 95.8–100% specificity, respectively, with substantial Cohen’s kappa agreement and statistically significant *p*-values. The developed assay showed potential as a molecular diagnostic tool for pathogen detection, with future application in herd-level screening warranting multicenter validation.

## 1. Introduction

Bovine paratuberculosis (ParaTB), caused by *Mycobacterium avium* subsp. *paratuberculosis* (MAP), and bovine tuberculosis (bTB), caused by *Mycobacterium bovis*, are significant zoonotic diseases that adversely affect livestock health and productivity and pose potential risks to public health [1]. ParaTB is widespread, with over 50% of dairy herds in the USA and Europe testing positive for MAP antibodies [2]. MAP establishes chronic infections by surviving inside macrophages through immune evasion mechanisms, resulting in delayed symptom onset and economic losses [3,4]. Similarly, *M. bovis* continues to spread in developing nations due to poor control measures, raising concerns of zoonotic tuberculosis comparable to Europe in the pre-pasteurization era [5,6]. Close contact with infected cattle further aggravates human exposure. 

Differentiation of MAP and *M. bovis* is difficult; although one belongs to the *Mycobacterium* avium complex (MAC) and the other belongs to the *Mycobacterium tuberculosis* complex (MTBC), the two species share 99.97–99.99% genetic similarity yet differ in pathogenicity, tissue tropism, and host preference; both are chronic wasting diseases that may present with non-specific manifestations, including progressive weight loss, poor body condition, reduced productivity, and chronic debility, particularly under field conditions [7]. Consequently, differential diagnosis may be challenging in endemic herds. The emergence of drug-resistant *M. bovis* strains due to inappropriate antibiotic exposure poses additional public health threats [8]. Low sensitivity or cross-reactivity limits conventional diagnostic methods like culture or simplex PCR. Molecular markers, including *IS900* and *F57* (MAP) [9,10], as well as *IS1081* and other genomic regions for MTBC detection, are frequently used in molecular diagnosis [11,12,13]. Duplex PCR, which amplifies two targets simultaneously, provides an accurate and specific alternative [14].

According to the World Organisation for Animal Health (WOAH), the diagnosis of bovine tuberculosis and paratuberculosis is based on a combination of immunological, bacteriological, and molecular methods, with culture remaining the reference standard despite its slow turnaround time and practical limitations. In endemic regions, both diseases can cause chronic weight loss, poor body condition, and reduced productivity, making differential diagnosis challenging under field conditions. Fecal sampling is a simple, non-invasive option for herd investigations and is routinely used for MAP detection, while *Mycobacterium bovis* may also be detected in the feces of animals with advanced infections. Therefore, a PCR assay that detects both pathogens in a single reaction offers a rapid and cost-effective alternative by reducing sample processing time, reagent use, and laboratory workload, making it particularly useful for preliminary screening in resource-limited settings. Bovine tuberculosis mainly affects the respiratory system and lymph nodes, but there have been reports of *Mycobacterium bovis* being shed in feces. This makes fecal sampling a beneficial, non-invasive way to screen herds. In resource-limited environments, where repeated or invasive sampling is not feasible, a single-sample diagnostic approach capable of detecting multiple chronic infections is highly advantageous. Therefore, the present study focuses on the development and optimization of a duplex PCR assay targeting *IS1081* for MTBC-associated detection of bovine tuberculosis and *IS900/F57* for MAP in blood and feces, offering a robust tool for accurate differential diagnosis and surveillance of two economically and zoonotically significant pathogens. In addition to fecal shedding, whole blood represents a practical diagnostic matrix for detecting systemic mycobacterial infections. During advanced or active stages of Johne’s disease and bovine tuberculosis, the detection of *IS900* and *IS1081* targets in peripheral whole blood indicates the presence of circulating mycobacterial DNA or phagocytosed fragments within leukocytes, rather than definitive evidence of viable bacteremia or active systemic dissemination [3,5]. Direct PCR on whole blood allows for the identification of systemic pathogen exposure alongside or prior to detectable mucosal shedding [15]. However, molecular detection in peripheral blood faces specific diagnostic limitations, including low and transient bacterial loads during subclinical or early infection stages, as well as the presence of potent PCR inhibitors such as heme, hemoglobin, and high host background DNA [15]. Incorporating paired blood and fecal samples into molecular diagnostic workflows provides a dual-matrix strategy to evaluate both systemic dissemination and mucosal shedding in clinically suspect animals [15].

## 2. Materials and Methods

### 2.1. Ethics

The present study was approved by the Institutional Biosafety Committee (IBSC) vide No. GLAU/IBSC/05/2023 and Registration No. GLAU231120211995. All animal handling and sample collection were performed by veterinarians in accordance with animal welfare regulations, with written informed consent obtained from cattle owners.

### 2.2. Bacterial Strains and DNA Extraction

*Mycobacterium avium* subsp. *paratuberculosis* strain ‘S 5’ (Indian Bison type), isolated from ruminants, was sourced from GLA University, Mathura, while the *M. bovis* AN5 strain and *M. smegmatis* were obtained from ICMR-National JALMA Institute, Agra. Genomic DNA was extracted from blood and fecal samples using the Qiagen DNA isolation kit and standardized laboratory protocol as per van Embden [16], with purity and concentration confirmed by Nanodrop and agarose gel electrophoresis.

#### 2.2.1. Method of Isolation of DNA from Blood Samples

A volume of 500 µL of whole blood was mixed with 1.0 mL of RBC lysis buffer (5 M NaCl, 2 M Tris, 3 M MgCl_2_; pH 7.6) by gentle inversion for 10 min, then incubated at room temperature (RT) for 10 min. Leukocytes were pelleted by centrifuging at 2500 rpm for 10 min. After discarding the supernatant, the pellet was resuspended into a slurry and washed repeatedly with RBC lysis buffer until a clean, white leukocyte pellet was obtained. This pellet was washed once with 1.0 mL of 1× PBS (pH 7.2), centrifuged at 2500 rpm for 5 min, and resuspended in 200 µL of WBC lysis buffer (5 M NaCl, 0.4 M EDTA, pH 8.0; 2 M Tris, pH 7.6; 10% Sodium Dodecyl Sulfate) containing 20 µL of proteinase K (10 mg/mL). The mixture was incubated at 56 °C for 4 h, allowed to cool to RT, and extracted with an equal volume of chloroform:isoamyl alcohol (24:1) by centrifuging at 10,000 rpm for 15 min at 4 °C. The upper aqueous phase was transferred to a new microcentrifuge tube, and genomic DNA was precipitated using 0.15 volumes of 3 M sodium acetate and 2.5 volumes of chilled absolute ethanol at −20 °C overnight. The precipitated DNA was pelleted at 10,000 rpm for 15 min at 4 °C, washed with 1.0 mL of 70% ethanol, air-dried, and dissolved in 25 µL of nuclease-free water.

#### 2.2.2. Isolation of DNA from Fecal Samples

Fecal material (2.0 g) was homogenized in 4.0 mL of sterile 1× PBS using a mortar and pestle. The homogenate was transferred to a 15 mL tube containing 8.0 mL of sterile 1× PBS and centrifuged at 3500 rpm for 45 min to concentrate bacterial cells. Then, the semi-solid middle layer was collected with a sterile swab and resuspended in 300 µL of 1× PBS in a 1.5 mL tube, followed by incubation with 40 µL of lysozyme (20 mg/mL) at 37 °C for 2 h under continuous shaking. After that, digestion was done with 20 µL of proteinase K (10 mg/mL) and 50 µL of 10% Sodium Dodecyl Sulfate at 56 °C for 2 h. Next, 64 µL of CTAB-NaCl was added, and the mixture was incubated at 65 °C for 30 min. An equal volume of chloroform:isoamyl alcohol (24:1) was added, and the sample was centrifuged at 10,000 rpm for 15 min at 4 °C. The upper aqueous phase was recovered, mixed with 0.6 volumes of isopropanol, and kept at −20 °C overnight to precipitate DNA. Finally, the DNA was pelleted by centrifugation at 10,000 rpm for 15 min at 4 °C, washed with 1.0 mL of 70% ethanol, air-dried, and dissolved in 25 µL of nuclease-free water.

### 2.3. Primer Validation

Primer specificity was further evaluated through in silico analysis using the NCBI GenBank database (BLASTn). The *IS900* primer set demonstrated high specificity for *Mycobacterium avium* subsp. *paratuberculosis*, whereas the *IS1081* primer set showed sequence conservation within members of the *Mycobacterium tuberculosis* complex (MTBC), including *M. bovis*. Therefore, *IS1081* was considered an MTBC-associated molecular target rather than an exclusive species-specific marker for *M. bovis*. No significant off-target amplification was identified against the non-MTBC sequences evaluated during the in silico screening.

### 2.4. PCR Amplification and Optimization

Species-specific primers were tested in simplex PCR using GoTaq Green Master Mix as described [17,18,19]. Gradient PCR was adopted for optimizing the annealing temperatures (50–64 °C). Thermal cycling was performed in a T100 Thermal Cycler (Bio-Rad) under the following conditions: initial denaturation at 95 °C for 5 min, 35 cycles of denaturation at 95 °C for 30 s, annealing at 61 °C for 30 s, and extension at 72 °C for 45 s, followed by final extension. Amplicons were visualized on 1.5% agarose gels. Compatible primer pairs identified in simplex assays were combined, generating duplex PCR targeting *IS1081* (*M. bovis*, an MTBC-associated target for bovine tuberculosis detection) + *IS900* (MAP-specific) genes (Table 1). Gradient PCR (58–62 °C) was performed to determine the optimal annealing conditions for each duplex assay. *M. smegmatis* DNA was used as a negative control for internal validation of duplex PCR.

### 2.5. Specificity and Limit of Detection (LOD)

Assay specificity was evaluated by cross-amplification of MAP primers with *M. bovis* DNA and vice versa, including no-template controls. Analytical sensitivity was evaluated using fecal samples spiked with serial ten-fold dilutions of MAP ‘S5’ and *M. bovis* ‘AN5’ ranging from 10 to 10^6^ CFU/g. Known bacterial concentrations were spiked into fecal samples prior to DNA extraction to simulate field matrix conditions. Each concentration was evaluated in triplicate under both simplex and duplex PCR conditions. The analytical limit of detection (LOD) was defined as the lowest bacterial concentration consistently detectable under the experimental conditions. The observed LOD was determined from the dilution series based on reproducible amplification, with inconsistent amplification at the lowest concentration (10 CFU/g) and consistent detection at 10^2^ CFU/g.

### 2.6. Preliminary Feasibility Assessment of Duplex PCR

A total of 114 specimens (57 blood and 57 fecal samples) from 57 suspected weak cattle with body condition score (BCS < 3 as inclusion criteria), weight loss, progressive emaciation, and persistent diarrhea in gausalas in the Mathura region were used for a preliminary feasibility evaluation. The Body Condition Score (BCS) was assessed using a standard 1 to 5 scale (ranging from 1 = emaciated to 5 = severely obese) as described by Edmonson [20]. Clinically healthy animals with BCS > 3 and without any history of chronic debility were excluded from the targeted suspect pool. Sampled animals were aged between 3 and 10 years, managed under intensive/semi-intensive herd housing conditions with communal feeding and watering facilities. The Indigenous ELISA was employed exclusively as a serological screening tool for MAP detection and utilized a semi-purified protoplasmic antigen, followed by sample-to-positive (S/P ratio) cutoff criteria. Indigenous ELISA, alongside *IS900* simplex PCR, served as a composite baseline to categorize MAP suspect cohorts. For MTBC-associated categorization, the *IS1081* simplex PCR was utilized. For diagnostic performance calculations, single-target simplex PCR assays (*IS900* for MAP; *IS1081* for MTBC) served as the primary reference comparator.

### 2.7. Statistical Analysis

Diagnostic performance of the duplex PCR (Evaluated Test B) was assessed relative to single-target simplex PCR assays (Reference Test A) using 2 × 2 contingency tables. Because an independent microbiological gold standard (such as culture) was not used, diagnostic performance metrics are reported as relative values, and categorical classifications indicate operational test agreement rather than definitive clinical infection status. The analytical LOD was determined experimentally from the serially spiked fecal samples as the lowest concentration showing consistent amplification under the tested PCR conditions. Categorical classifications were defined based on paired test outcomes: True Positive (TP): sample tested positive by both Reference Test A and Evaluated Test B; True Negative (TN): sample tested negative by both Reference Test A and Evaluated Test B; False Positive (FP): sample tested negative by Reference Test A but positive by Evaluated Test B; and False Negative (FN): sample tested positive by Reference Test A but negative by Evaluated Test B. Relative diagnostic sensitivity and relative diagnostic specificity were calculated using the following equations: relative sensitivity is TP/(TP + FN) × 100, whereas relative specificity is TN/(TN + FP) × 100. Precision metrics were reported with numerical 95% confidence intervals (95% CI). Inter-assay agreement was quantified using Cohen’s kappa coefficient (kappa) and interpreted according to standard benchmarks.

## 3. Results

**Figure 1:**
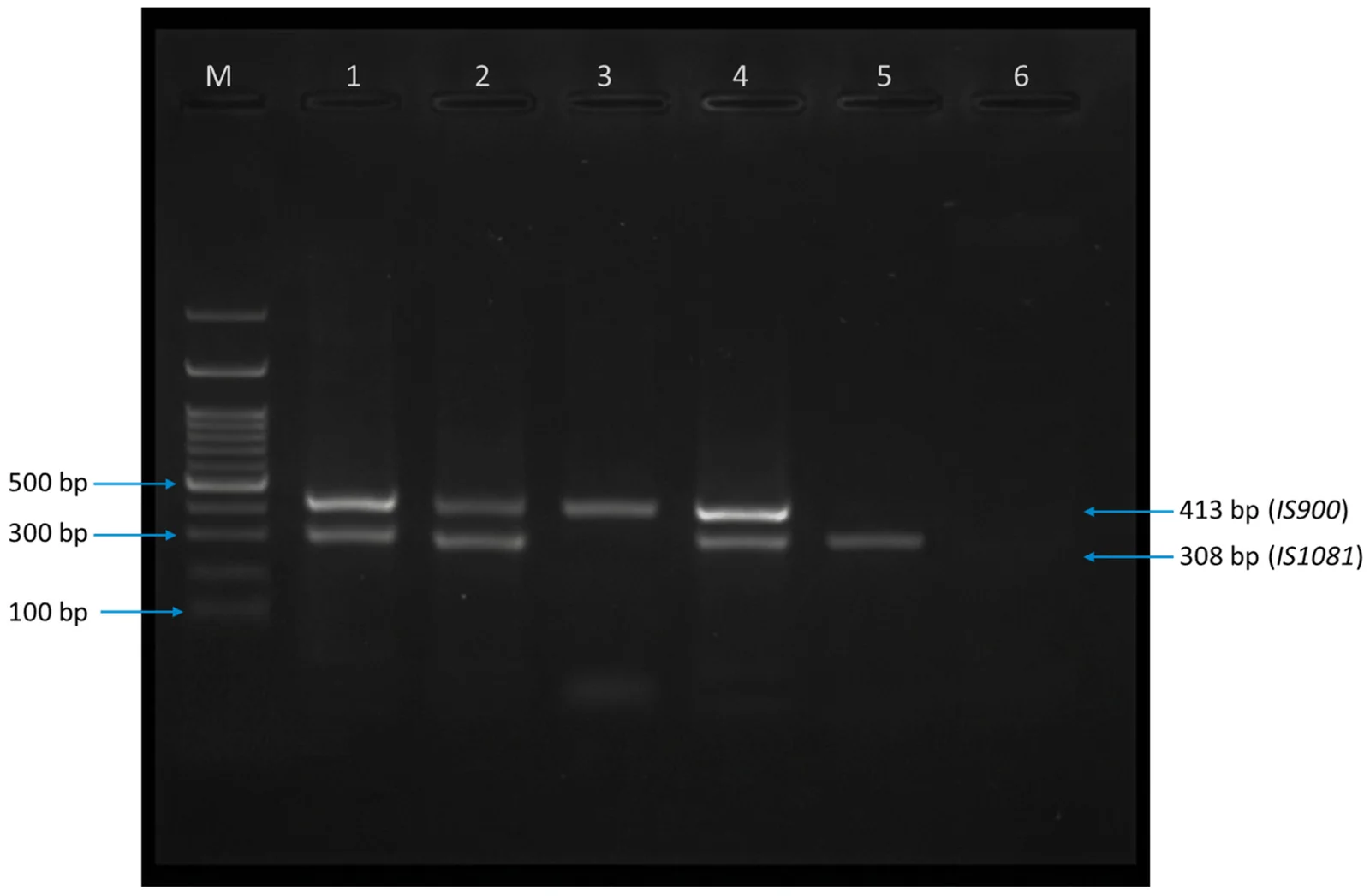
Molecular identification of *M. paratuberculosis* and MTBC-associated DNA targeting *IS900* and *IS1081* genes using duplex PCR. Lane M: 100 bp Ladder; Lane 1: Positive control (DNAs of both *M. paraTB* and *M. bovis*); Lanes 2 and 4: Duplex PCR targeting *IS900* (*M. paraTB*) and *IS1081* (MTBC-associated *M. bovis*) genes; Lane 3: Simplex PCR targeting *M. paraTB IS900* gene; Lane 5: Simplex PCR targeting MTBC-associated *M. bovis IS1081* gene; Lane 6: Negative control.

All selected primer pairs were initially evaluated in simplex PCR to verify amplification efficiency. Among the MTBC-associated *M. bovis* targets (*RD9*, *IS1081*, and *16S rRNA*), the *IS1081* primer pair consistently produced the expected 308 bp fragment, whereas the *M. avium* subsp. *paratuberculosis IS900* primer pair generated a distinct and reproducible amplicon of 413 bp (Table 1). Gradient PCR revealed that an annealing temperature of 60 °C yielded the sharpest and most specific bands, confirming optimal conditions for duplex reactions. The duplex PCR assay simultaneously amplified *IS900* and *IS1081* targets, producing two distinct bands of 413 bp (*M. paratuberculosis*) and 308 bp (MTBC-associated detection of bovine tuberculosis) (Figure 1). The presence of both bands in single reactions verified the capability of the duplex assays to detect both pathogens concurrently.

Specificity testing demonstrated no cross-reactivity: the MAP primer did not amplify *M. bovis* DNA and vice versa, while *M. smegmatis* and negative controls remained amplification-free. In simplex PCR, both *IS900* and *IS1081* targets were consistently detected up to 10^2^ CFU/g, which remained unchanged upon transition to duplex PCR conditions. No observable loss in band intensity or amplification efficiency was noted in duplex reactions compared to individual assays, suggesting minimal primer–primer interference under optimized conditions (60 °C annealing temperature and adjusted primer concentrations). Analytical sensitivity testing using spiked fecal samples demonstrated consistent detection at 10^2^ CFU/g for both *IS900* and *IS1081* targets in simplex PCR, with the same detection threshold observed under duplex PCR conditions. Thus, the experimentally determined LOD was 10^2^ CFU/g for both pathogens in both assay formats. Amplification at 10 CFU/g was inconsistent, indicating that this concentration was below the reliable detection threshold of the conventional PCR assay. Moreover, the preliminary feasibility assessment using 114 categorized blood and fecal samples from 57 clinically suspected cattle in local gausalas serves as preliminary proof-of-concept for a duplex PCR platform evaluated under pilot field conditions. Using single-target *IS900* and *IS1081* PCRs as reference comparators, the duplex assay achieved 63.6–100% sensitivity and 95.8–100% specificity, respectively, with moderate to substantial kappa agreement (Table 2). The Cohen’s kappa (κ) values, calculated with a 95% confidence interval, were interpreted according to standard values: <0.20 indicated poor agreement, 0.21–0.40 fair agreement, 0.41–0.60 moderate agreement, 0.61–0.80 substantial (good) agreement, and 0.81–1.00 almost perfect agreement.

## 4. Discussion

The results confirm that *IS1081* serves as a robust molecular marker for MTBC detection in bovine samples, while *IS900* remains a reliable and specific target for *M. avium* subsp. *paratuberculosis* detection. The use of *IS1081* was based on its established utility for MTBC detection and its particular relevance to bovine tuberculosis surveillance, where *M. bovis* represents the principal pathogen of concern in cattle. The detection of *IS900* and *IS1081* genomic targets in peripheral whole blood indicates the presence of circulating mycobacterial DNA or phagocytosed mycobacterial fragments within circulating leukocytes. However, PCR-based detection of target DNA in blood should not be interpreted as definitive evidence of viable bacteremia or active systemic dissemination, as DNA can persist following immune-mediated bacterial destruction or intermittent hematogenous clearance.

Although other MTBC members, including *M. caprae*, can cause bovine tuberculosis in cattle, their epidemiological contribution varies substantially by region. In the Indian bovine context, *M. bovis* is considered the principal bovine tuberculosis-associated MTBC pathogen, whereas reports of *M. caprae* are comparatively limited. These findings align with previous studies reporting *IS1081* as a conserved element in MTBC-associated *M. bovis* and *IS900/F57* as highly specific MAP markers in formalin-fixed, paraffin-embedded bovine tissues [10,14,21]. Although recent studies have reported certain limitations in commonly used *IS900* primer sets, including sequence variability and partial mismatches, the selected primers in the present study demonstrated consistent amplification, high specificity, and no cross-reactivity under optimized conditions, supporting their continued utility in conventional PCR-based diagnostics. The absence of cross-amplification demonstrates the high specificity and diagnostic reliability of the developed duplex PCR assay. Compared to culture-based identification, which is slow, labor-intensive, and prone to contamination, the duplex PCR provides accurate differentiation of MTBC-associated *M. bovis* and *M. paratuberculosis*. Preliminary evaluation of the duplex PCR indicates excellent diagnostic kappa agreement with established molecular methods and demonstrates the assay’s suitability for direct detection of pathogens in fecal samples—a challenging matrix due to PCR inhibitors and variable bacterial load.

The observed limit of detection (10^2^ CFU/g) aligns with previously reported sensitivity ranges for conventional PCR and real-time PCR assays [10,15]. Although digital and triplex PCR approaches [8] may achieve higher sensitivity and quantification accuracy, their requirement for advanced instrumentation limits their utility in low-resource, field, or routine diagnostic laboratories [9]. The duplex PCR assay demonstrated potential for simultaneous detection of MTBC-associated *M. bovis* and MAP, with preliminary pilot field evaluation supporting its feasibility and warranting further multicentre validation for broader diagnostic application in endemic regions.

The rationale for combining MTBC-associated *Mycobacterium bovis* and *Mycobacterium avium* subsp. *paratuberculosis* in a single assay is based on their co-endemicity, overlapping clinical presentation, and shared impact on livestock productivity and public health. Although bovine tuberculosis (bTB) and paratuberculosis (Johne’s disease) share similar chronic clinical manifestations such as progressive emaciation and reduced milk yield, their epidemiological, regulatory, and public health implications differ fundamentally, making precise differential diagnosis critical [5,22]. *Mycobacterium bovis* is a high-consequence zoonotic pathogen subject to strict regulatory control, mandatory reporting, and test-and-slaughter or quarantine policies in many jurisdictions to prevent transmission to humans and secondary livestock hosts [3,10]. In contrast, management of *M. avium* subsp. *paratuberculosis* (MAP) primarily relies on long-term herd-level control, hygienic calf-rearing, and culling of heavy shedders to minimize economic losses rather than immediate government-mandated herd depopulation [2,10]. Distinguishing between the two pathogens in a single, rapid assay prevents misclassification, avoids inappropriate herd management decisions, resolves skin-test cross-reactivity in co-infected herds, and provides immediate, actionable data for public health biosecurity and targeted surveillance [3,23].

Unlike acute enteric pathogens, both infections are chronic and require long-term management. Fecal samples, although optimal for MAP detection, also provide a non-invasive matrix for detecting MTBC-associated *M. bovis* at the herd level, particularly in resource-limited settings. The present study has a few limitations that should be considered when interpreting the findings. The preliminary feasibility assessment was conducted on 57 clinically suspected cattle from a single geographic region, which may limit the broader applicability of the results. While assay specificity was confirmed using *M. smegmatis* and non-template controls, the analytical specificity panel did not include a broader spectrum of environmental non-tuberculous mycobacteria (NTM) or common enteric co-pathogens. In addition, culture, regarded as the reference gold standard, was not included because of its slow growth and long turnaround time; pilot field evaluation relied on composite molecular and serological reference methods (*IS900*/*IS1081* single-target PCRs and Indigenous ELISA) rather than an independent microbiological gold standard. This pilot-scale field testing highlights key outcomes and explicitly identifies necessary multi-center validation steps required prior to potential routine diagnostics. In fecal specimens, the lower *IS900* positivity observed with the duplex PCR indicates reduced performance in complex matrices compared to single-target simplex PCR. Potential mechanisms such as residual PCR inhibitors, primer competition, or low target DNA concentrations were not directly evaluated in this study. This reduced detection efficiency in fecal samples represents a recognized limitation of the current assay, highlighting the need for future reaction optimization and enhanced DNA extraction. Although the experimentally determined LOD was 10^2^ CFU/g for both simplex and duplex PCR, the analytical sensitivity assessment was based on triplicate testing of a defined serial dilution series. A more comprehensive LOD_95_ analysis using a larger number of replicates across multiple concentrations, together with confidence intervals around the regression-derived detection probability, would provide a more statistically robust estimate of analytical sensitivity. Future studies should therefore include expanded replicate testing and formal probit or logistic regression-based LOD_95_ estimation. This preliminary proof-of-concept study reflects single-center pilot testing. Future studies involving larger and geographically diverse animal populations, culture-based confirmation, and comparisons with advanced molecular techniques such as digital droplet PCR are essential to fully establish the diagnostic accuracy, inter-laboratory reproducibility, and broad field applicability of this duplex PCR assay prior to routine diagnostic implementation.

## Figures and Tables

**Table 1:** Optimization and evaluation of duplex PCR for simultaneous detection of MTBC-associated bovine tuberculosis and *Mycobacterium avium* subsp. *paratuberculosis*.

Primers A (Target Gene)	Primers B (Target Gene)	Optimized PCR Reaction Mix and Annealing Conditions (Total Volume 15 μL)	Product Sizes
P90B & P91B [16] (gene *IS900*) P90B-Forward GAAGGGTGTTCGGGGCCGTCGCTTAG P91B- Reverse GGCGTTGAGGTCGATCGCCCACGTGC	IS1081F & R [17] (gene *IS1081*) BW-6F-5′-CGACACCGA GCAGCT TCTGGCTG 3′ BW-7 R-5′-GTCGGCACCACG CTGGCTAGTG-3′	Go Green Taq MM + FP & RP (MAP) 0.15 μL + FP & RP (Mb) 0.3 μL (10 pmol) MAP (+ve) control DNA 2 μL + Mb (+ve) control DNA 3 μL Annealing temperature: 60 °C	413 bp and 308 bp

MM—master mix, FP—forward primer, RP—reverse primer, MAP—*Mycobacterium paratuberculosis*, Mb—MTBC-associated *M. bovis*, (+ve)—positive control DNA.

**Table 2:** Comparative diagnostic performance and agreement between single-target reference assays and the duplex PCR assay in 114 field specimens from 57 suspected weak cattle.

Positives in Reference Test A		Positives in Evaluated Test B		Positives in Both Tests A & B	Cohen’s Kappa (95% CI)	Relative Sensitivity (95% CI)	Relative Specificity (95% CI)
Fecal samples (n = 57)							
IS1081 PCR	07	Duplex PCR	07	07	1.000 (1.000–1.000)	100.0% (59.0–100.0)	100.0% (92.9–100.0)
IS900 PCR	11	Duplex PCR	07	07	0.739 (0.472–1.000)	63.6% (30.8–89.1)	100.0% (92.3–100.0)
Blood samples (n = 57)							
IS1081 PCR	09	Duplex PCR	08	06	0.702 (0.500–0.904)	66.7% (29.9–92.5)	95.8% (85.7–99.5)
IS900 PCR	09	Duplex PCR	08	07	0.823 (0.664–0.982)	77.8% (40.0–97.2)	97.9% (85.7–99.5)

Test A and Test B represent the diagnostic assays being evaluated in pairwise combination. Test combinations evaluate agreement between single-target molecular assays (IS1081 PCR for MTBC-associated amplification of bovine-associated tuberculosis; IS900 PCR for *M. paratuberculosis*) and the optimized duplex PCR. CI—confidence interval.

## Data Availability

The data supporting the findings of this study are available from the corresponding author upon request.
